# Identification of significant climatic risk factors and machine learning models in dengue outbreak prediction

**DOI:** 10.1186/s12911-021-01493-y

**Published:** 2021-04-30

**Authors:** Felestin Yavari Nejad, Kasturi Dewi Varathan

**Affiliations:** Department of Information System, Faculty of Computer Science and Information Technology, University of Malaya, Kuala Lumpur, Malaysia

**Keywords:** Risk factor, Dengue, Outbreak prediction model, TempeRain factor

## Abstract

**Background:**

Dengue fever is a widespread viral disease and one of the world’s major pandemic vector-borne infections, causing serious hazard to humanity. The World Health Organisation (WHO) reported that the incidence of dengue fever has increased dramatically across the world in recent decades. WHO currently estimates an annual incidence of 50–100 million dengue infections worldwide. To date, no tested vaccine or treatment is available to stop or prevent dengue fever. Thus, the importance of predicting dengue outbreaks is significant. The current issue that should be addressed in dengue outbreak prediction is accuracy. A limited number of studies have conducted an in-depth analysis of climate factors in dengue outbreak prediction.

**Methods:**

The most important climatic factors that contribute to dengue outbreaks were identified in the current work. Correlation analyses were performed in order to determine these factors and these factors were used as input parameters for machine learning models. Top five machine learning classification models (Bayes network (BN) models, support vector machine (SVM), RBF tree, decision table and naive Bayes) were chosen based on past research. The models were then tested and evaluated on the basis of 4-year data (January 2010 to December 2013) collected in Malaysia.

**Results:**

This research has two major contributions. A new risk factor, called the TempeRain factor (TRF), was identified and used as an input parameter for the model of dengue outbreak prediction. Moreover, TRF was applied to demonstrate its strong impact on dengue outbreaks. Experimental results showed that the Bayes Network model with the new meteorological risk factor identified in this study increased accuracy to 92.35% for predicting dengue outbreaks.

**Conclusions:**

This research explored the factors used in dengue outbreak prediction systems. The major contribution of this study is identifying new significant factors that contribute to dengue outbreak prediction. From the evaluation result, we obtained a significant improvement in the accuracy of a machine learning model for dengue outbreak prediction.

## Background

Pandemic infectious diseases are spreading in many geographical areas. The World Health Organisation (WHO) has reported that dengue fever is one of the most important mosquito-borne and deadliest infectious diseases which have been caused by the dengue virus. Accordingly, this disease is a threat and poses severe risk to human populations in tropical and subtropical regions [1–7]. Member states in the three WHO regions regularly reported an increase in the annual number of cases from 2.2 million in 2010 to 3.2 million in 2015 [8]. A recent study from WHO indicated that 390 million dengue infections occur annually (95% credible interval of 284–528 million); among which, 96 million (67–136 million) are manifested clinically with any severity of the disease [9, 10]. There is a research that works on how dengue has changed global threat by vector-borne disease and will help decision-makers worldwide to better prepare for and respond to future changes in dengue risk for the years 2020, 2050 and 2080 [11].

As of December 2018, the Ministry of Health (MOH) of Malaysia has recorded approximately 80,615 dengue cases with 147 deaths compared with 19,884 cases in December 2011 with 36 deaths [12].The number of cases increased approximately fourfold. By the end of March 2019, 39,805 cases of dengue with 64 deaths were reported in Malaysia compared with 16,917 cases with 34 deaths in March 2018 [13].

The alternative solutions would be to prevent and control the outbreak of this disease. One of the ways in achieving this is by having a good prediction on the existence of the outbreak. This kind of predictions helps the higher authorities as well as individuals to devise plans and handle the situation in an efficient and effective manner [14, 15]. The accuracy of a prediction system for outbreaks is the primary and important concern for controlling dengue fever [14]. Therefore, establishing related risk factors is critical for prediction systems [16]. Given that climate factors play a key role in this disease, identifying the relation between weather information and dengue outbreak incidence is a major task in establishing an accurate prediction system for future outbreaks [17–19]. In the current study, important climatic risk factors, such as temperature, relative humidity and rainfall amount, were examined. The current accuracy for prediction systems based on climate factors ranges from 82.39 to 90.5% [16, 20–25].

This research aims to describe the dengue prediction system accuracy and the level of risk factors that contribute to a dengue outbreak prediction system and identify the associations amongst new climate risk factors. The detailed factors are then used as inputs for predicting dengue outbreaks.

## Related works

Various early warning and monitoring systems are currently implemented to monitor dengue outbreaks worldwide. Dengue prediction models have been previously investigated, but some of these models still exhibit limitations in achieving high accuracy in dengue outbreak prediction [14, 15]. Different models and techniques have been integrated into the design of several models for predicting dengue outbreaks. A number of studies have also established prediction models for dengue outbreaks using artificial neural networks [16].

Hybrid models have been used in outbreak prediction research. A hybrid model is an example of an integrated model, and many models based on genetic algorithms are available to determine the weight in a neural network model [14, 17–19, 25, 26]. In Singapore, researchers found significant correlated dengue cases with climatic variables by using a Poisson regression model [27]. One researcher [22] developed a dengue outbreak prediction system in Singapore and obtained 90% accuracy. There are research that was established by using decision tree in prediction system [23]. One research in Indonesia was concerned with dengue outbreak prediction using a GIS-based early warning system [20]. Another study from the National Taipei University of Technology used C-support vector classification to forecast dengue fever epidemics in Taiwan, and the accuracy of radial basis function (RBF) model was 90.5% [21].

Vulnerability maps of dengue incidences have been generated in Malaysia, resulting in the development and implementation of visualised and predictive modelling using geographic information systems (GIS) for dengue fever in Selangor, Malaysia [28]. There are different models of dengue outbreak prediction systems in Malaysia have achieved different accuracies [16, 25].In 2015, [29] predicted localised dengue incidences in Malaysia using an ensemble system for identification and found that ensemble models exhibit better prediction power than a single model [29].The prediction of dengue outbreaks is crucial worldwide because this infectious disease remains as a major issue in many countries [14, 26, 30, 31]. Table 1 lists studies on different models of dengue outbreak prediction with distinct climatic risk factors. The asterisk (*) in the columns of the table denotes the risk factors used in different studies.

**Table 1:** Risk factors used in different researches for dengue outbreak prediction models from 2005 to 2018

References	Technique	Year	Geographical data used	Temperature			Humidity	Rainfall					Mean
				Min	Avg	Max	Relative (mean)	Cumulative rainfall	Total rainfall	Max 24-h rainfall	Max 1-H rainfall	Bi-weekly	
[32]	Wavelet coherence analysis/quasi-Poisson regression combined with distributed lag nonlinear model (DLNM)	2018	Philippines		*			*					
[33]	Generalized linear model	2018	Bangladesh		*		*		*				
[34]	Negative binomial regression (NBR)/generalized estimating equation (GEE)	2017	Vietnam		*				*				
[35]	Artificial neural network (ANN)	2016	Philippine		*		*		*				
[36]	Distributed lag non-linear models (DLNM)/generalised estimating equation models (GEE)	2016	China	*		*	*						*
[37]	Spearman rank correlation/distributed lag non-linear model (DLNM)	2014	Singapore	*	*	*	*		*				*
[38]	Distributed lag nonlinear model (DLNM) and Markov random fields	2014	Taiwan	*	*	*			*	*	*	*	
[39]	Generalized additive model (GAM)	2014	Europe	*		*	*		*				
[40]	Generalized additive model (GAM)	2013	Mexico	*		*			*				
[41]	Poisson generalized additive model/distributed non-linear lag model (DLMN)	2013	Malaysia,	*	*	*	*					*	*
[22]	Poisson multivariate regression models	2013	Singapore		*			*					
[42]	Autoregressive integrated moving average (ARIMA)	2013	Malaysia		*		*		*				
[43]	Poisson multivariate regression	2012	Singapore		*			*					
[44]	Spearman's rank correlation coefficient (SRCC)	2012	Singapore	*			*		*				
[3]	Vector–host transmission model	2012	Taiwan	*		*	*		*				
[14]	Neural network and genetic algorithm	2012	Malaysia						*				
[45]	Generalised linear model (GLM)/Bayesian framework using Markov chain Monte Carlo (MCMC)	2011	Brazil		*		*		*				
[16]	Artificial neural networks (ANN)	2010	Singapore		*		*		*				
[46]	Multiple regression and discriminant analysis techniques/Peirce skill score	2010	Indonesia	*	*	*	*		*				
[47]	Artificial neural networks (ANN)	2009	Turkey		*		*		*				
[48]	Entropy and artificial neural network	2008	Thailand	*	*	*	*		*				
[49]	Kolmogorov-Sminov test/Pearson’s correlation coefficient/stepwise regression techniques	2005	Thailand	*	*	*	*		*				
	Total	11	16	10	15	3	17	1	1	2	3		

Most studies on dengue fever were conducted in Asian countries, such as Malaysia, Singapore, Taiwan, Indonesia, Bangladesh and Thailand, are critical areas for dengue fever. Most studies have shown that temperature and rainfall directly and significantly affect dengue outbreaks [15, 18, 25, 26, 30, 31].

Moreover, changing climatic factors, such as increasing temperature, rainfall and humidity, are the most influential driving forces of dengue virus transmission [31]. One study correlated dengue cases with climatic variables in the city of Singapore and the model for dengue cases was considered the dependent variable; meanwhile, climatic variables, such as rainfall, maximum and minimum temperatures and relative humidity, were considered independent variables [27]. On the basis of the grade of each risk factor used in the 22 references listed in Table 1, most studies primarily used total rainfall (17 studies), average temperature (16 studies), relative humidity (15 studies), minimum temperature (11 studies) and maximum temperature (10 studies) as inputs of prediction models. However, none of the studies focused on the detailed analysis of the factors nor investigated the detailed relationship that can exist amongst factors.

## Methods

**Fig. 1:**
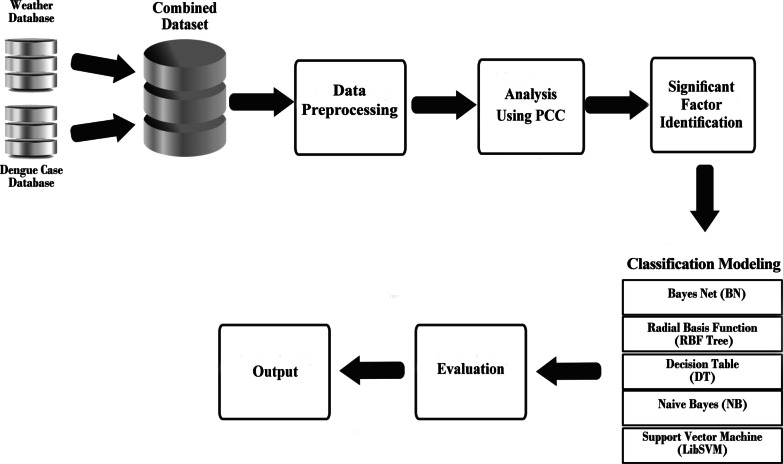
Conceptual framework for identifying significant climate factors in dengue outbreak prediction

This section explains the methodology used for this research, including the dataset used, the analysis process, the newly identified integrated input factors, the evaluation with machine learning models and the evaluation method. Figure 1 illustrates the conceptual framework of our research.

### Dataset

Data are retrieved from two official sources. The dengue fever incident and confirmed cases has published weekly on Ministry of Health Malaysia (MOH) portal and the report of location and number of dengue confirmed case is available and accessible on weekly basis from [12]. In order to access each of the files, the following link provides the access. However, the report number based on the weeks must be stated explicitly.

http://www.moh.gov.my/index.php/database_stores/attach_download/337/report number

For example to retrieve the report no. 234 (week10, 2012):

http://www.moh.gov.my/index.php/database_stores/attach_download/337/234

Besides that, this report can also be obtained via a simple search in any search engine by using the following search terms together with required week number and year:

“SITUASI SEMASA DEMAM DENGGI DI MALAYSIA Bagi Minggu *week number/year”*

*English Translation:*

Situation of Dengue Fever in Malaysia for *week number/year*

Moreover, the climatic data are obtained from Malaysian Meteorological Department (MMD) [50]. However, the processed data is available upon reasonable request from the authors.

**Fig. 2:**
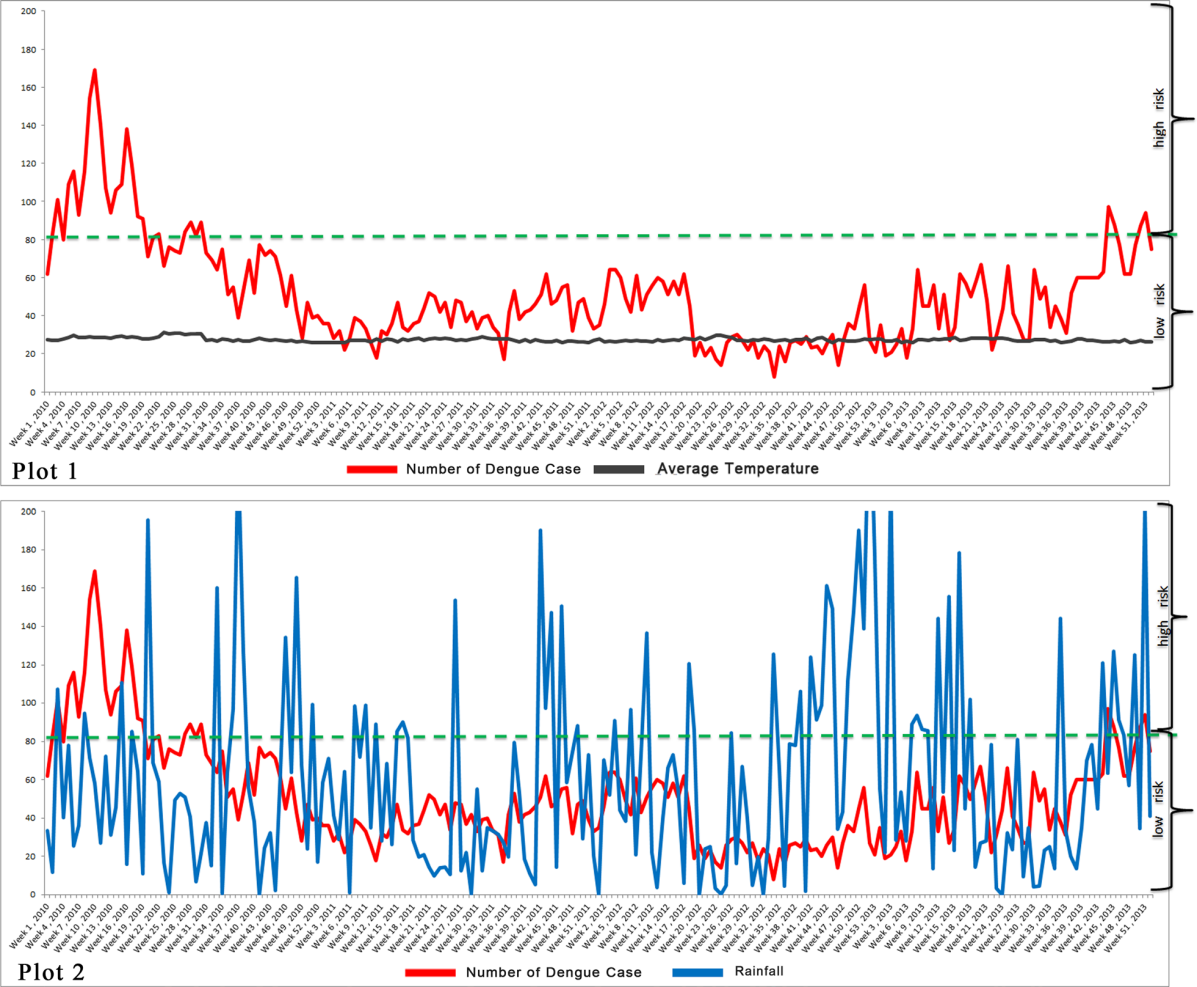
Weekly incidence of dengue with average temperature and rainfall from January 2010 to December 2013 (week 1 to week 209)

Data were collected from two sources. We obtained weekly data on dengue confirmed cases based on two federal territories, namely, Kuala Lumpur (Wilayah Persekutuan Kuala Lumpur) and Putrajaya, from January 2010 to December 2013. The weather data of Kuala Lumpur and Putrajaya were retrieved from Malaysian Meteorological Department (MMD) for the period of January 2010 to December 2013 [50]. Thus, 209 weeks of confirmed dengue cases and meteorological data were evaluated in this study. However, approximately 8% of the data were missing in the MMD datasheets for the study period. Thus, we obtained the missing data for this period from the US Weather Channel Interactive (https://weather.com), which also provides Malaysian meteorological data. The data were fitted simultaneously with the Putrajaya–Cyberjaya Station in Malaysia. Only minimum temperature, maximum temperature, average temperature, minimum humidity and rainfall were selected because many studies have emphasised that these factors are the most important risk factors for dengue outbreak prediction models, as shown in Table 1. Figure 2 illustrates two plots of data from January 2010 to December 2013.

The data are combined and cleaned accordingly. The preprocessed data are analysed, and new detailed factors are identified. The factors are then integrated and fed as integrated inputs to different machine learning models and evaluated. The following sections provide a detailed description of each process involved in this framework.

### Analysis

Weather data from MMD provide daily weather information, and the incidence of dengue cases is published weekly by MOH. Thus, data were normalised and classified into two levels namely, ‘low risk’ and ‘high risk’, on a weekly basis [51] Weather and meteorological factors play important roles in the incidence of dengue fever. Thus, the dataset was analysed, and the relationship between the incidence of dengue cases and weather information was determined every week using the Pearson correlation coefficient (PCC) [52].

1 $${\text{R}} = \frac{{\sum {{\text{x}}_{{\text{i}}} {\text{y}}_{{\text{i}}} } - \frac{{\sum {{\text{x}}_{{\text{i}}} } \sum {{\text{y}}_{{\text{i}}} } }}{{\text{N}}}}}{{\sqrt {\left( {\sum {{\text{x}}_{{\text{i}}}^{2} - \frac{{\left( {\sum {{\text{x}}_{{\text{i}}} } } \right)^{2} }}{{\text{N}}}} } \right)\left( {\sum {{\text{y}}_{{\text{i}}}^{2} - \frac{{\left( {\sum {{\text{y}}_{{\text{i}}} } } \right)^{2} }}{{\text{N}}}} } \right)} }}$$

### Identification of significant factors

The most significant climate factors were identified based on the correlation analysis of the dataset, as shown in Table 2. The analysis result indicated that the highest correlation exists between minimum temperature and dengue incidence, followed by cumulative rainfall and the incidence of dengue cases determined in different weeks.

**Table 2:** Correlation between dengue incidence cases and climate factors

Temperature			Mean relative humidity	Rainfall
Minimum temperature	Mean temperature	Maximum temperature		
0.447	0.339	0.316	− 0.176	− 0.020

Minimum temperature and daily rainfall are the most significant dengue weather-based risk factors [36, 53–56]. The average minimum temperature can be calculated as follows (Eq. 2):

2 $${\text{Average}}\;{\text{Min}}\;{\text{Temperature}}\;{\text{Week(i)}} = \frac{{{\text{Minimmum}}\;{\text{Temperature}}\;{\text{(Current}}\;{\text{Week)}} + \sum\nolimits_{n = 0}^{5} {{\text{Min}}\;{\text{Temperature}}\;[{\text{Week}}({\text{i}} - {\text{n}})]} }}{6}$$

where *i* is the number of weeks from which the average minimum temperature and [Week(i − n)] is the minimum temperature of the prior weeks to the current week plus minimum temperature of current week [n = 0]. To find average, the result divided by 6 [5 weeks before plus current week].

The cumulative rainfall for week *i* can be calculated using Eq. 3, as follows:

3 $${\text{Cumulative}}\;{\text{Rain}}\,{\text{fall}}\;{\text{Week(i) = }}\sum\limits_{{\text{n = 0}}}^{1} {{\text{[Total}}\;{\text{Rainfall}}\;{\text{Week(i}} - {\text{n)]}}}$$

where *i* is the desired week from which the total rainfall will be calculated, cumulative rainfall week (i) is the final calculation and week (i − n) is the week prior to week (n).

Table 3 provides the PCCs between the weather variables and the incidence of dengue cases. The underlined and highlighted high positive numbers showed the highest correlation and coefficients between weather parameters and the incidence of dengue fever. Table 3 presents the results for 7 weeks prior to the current week and the optimum value for the average minimum temperature (0.499).

**Table 3:** Pearson correlation coefficient (PCC) between climatic factors and incidence of dengue cases

	Average minimum temperature	Cumulative rainfall
Current week	0.447	− 0.0201
1 Week prior	0.465	0.0065
2 Week prior	0.480	0.0071
3 Week prior	0.494	− 0.0005
4 Week prior	0.498	− 0.0123
5 Week prior	0.499	− 0.0139
6 Week prior	0.489	− 0.0045
7 Week prior	0.476	0.0020

The highest value for cumulative rainfall (0.0071) was obtained for 2 weeks prior to the current week (Table 3).

**Fig. 3:**
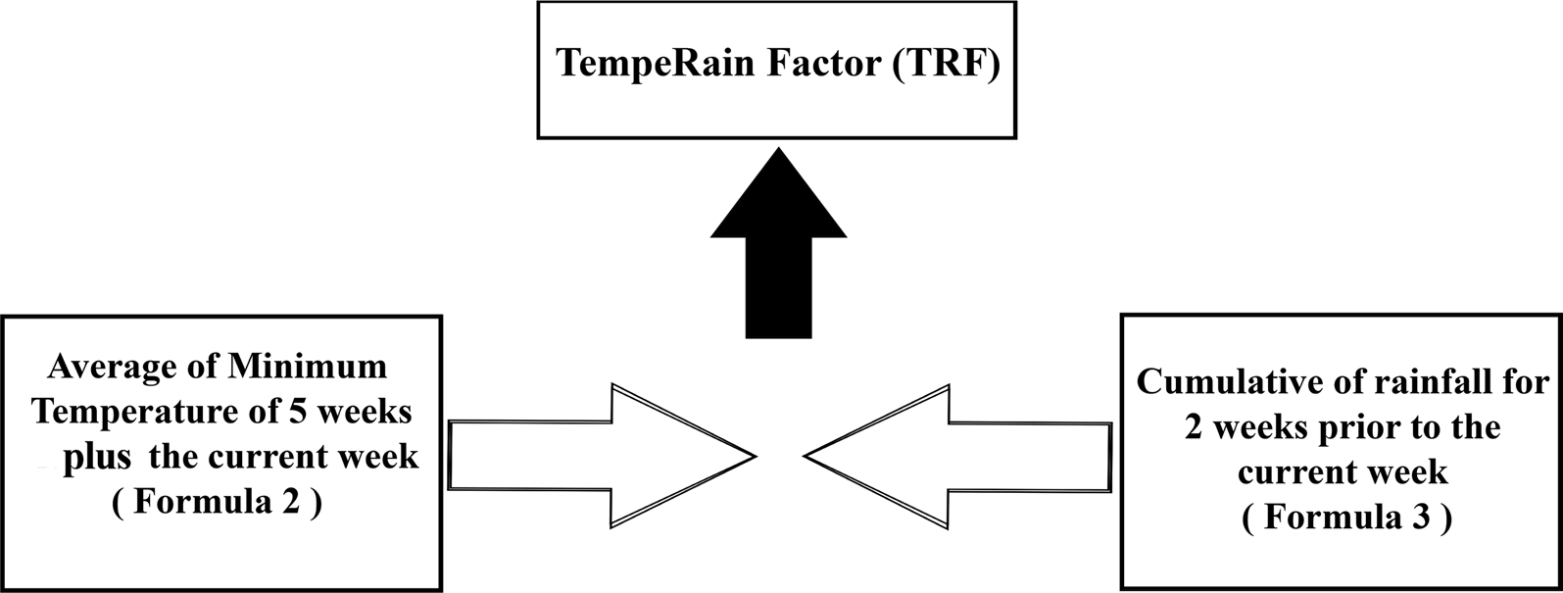
Components of TempeRain factor (TRF)

Thus, the average minimum temperature of Week 5 (plus the current week) and the cumulative rainfall for Week 2 (prior to the current week) exhibit high correlation with dengue cases in accordance with the correlation analysis. The two factors will be regarded as TRF and used as input parameters for dengue outbreak risk level prediction. The combination of factors is shown in Fig. 3.

The cumulative rainfall for 2 weeks prior to the current week is identified as a significant factor because it coincides with the life cycle of an Aedes aegypti mosquito, i.e. approximately 2 weeks. Thus, this shows that there is a possibility that it may happen right after an A. aegypti mosquito completes its life cycle and becomes an adult [38, 53–58].

### Prediction using machine learning models

Once significant factors have been identified, the research proceeded towards predicting the risk incidence level of dengue fever. We considered high risk as existence of dengue outbreak and low risk as no outbreak. To predict this level, we tested five machine learning models using input factors with and without TRF. Table 4 provides the detailed input factors and descriptions.

**Table 4:** List of input factors used in prediction model with identified factors (TRF) and without TRF

Input factors without TRF		Input factors with TRF	
Type	Parameter description	Type	Parameter description
Weather factors	Minimum temperature (°C)	Weather factors	
	Mean temperature (°C)		Mean temperature (°C)
	Maximum temperature (°C)		Maximum temperature (°C)
	Mean relative humidity (%)		Mean relative humidity (%)
	Cumulative of rainfall (mm)		
		TRF factors	Average of minimum temperature 5 weeks plus current week (°C)
			Cumulative of rainfall for 2 weeks prior to the current week (mm)

On the basis of the high accuracies obtained [21, 59], we selected Bayes network (BN) models, support vector machine (SVM), RBF tree, decision table and naive Bayes to evaluate the factors using WEKA version 3.8.0 [60]. We used the cross-validation (tenfold) technique to evaluate the models.

### Evaluation metrics

We can evaluate the performance of classifiers on the basis of several accuracy measures and parameters. Moreover, some accuracy and error measures are used to determine the distance between the predicted and the actual known values [61]. In this study, we used the accuracy metric to measure the performance of the classifiers [52, 62, 63]. Equation 4 shows how accuracy is calculated.

4 $$\mathrm{Accuracy}=100* \frac{(\mathrm{TP}+\mathrm{TN})}{(\mathrm{TP}+\mathrm{FP}+\mathrm{TN}+\mathrm{FN})}$$

## Results

Table 5 presents the results from five machine learning models with and without TRF inputs. Improved results and reduced errors were obtained using the weather data (as external risk factors for a dengue fever outbreak prediction model) by applying machine learning models (as data analysers) and adding newly identified factors (TRF).

**Table 5:** Machine learning classifier models using cross-validation (tenfold) with TempeRain factor (TRF)

Models	Accuracy (%)
Bayes net	
With TRF	**92.35**
Without TRF	91.39
SVM	
With TRF	88.04
Without TRF	88.00
RBF tree	
With TRF	89.47
Without TRF	89.47
Decision table	
With TRF	90.41
Without TRF	89.95
Naive Bayes	
With TRF	89.4737
Without TRF	88.9952

Thus, the proposed factors and machine learning model are beneficial for predicting the dengue risk level. The results also showed that models with TRF achieved slightly higher accuracies compared with those without TRF. The highest accuracy was obtained by the BN classifier with TRF (92.35%).

Other studies exhibit different accuracies based on their own private databases, which consist of data collected from patients in hospitals, compared with our research area [20, 23, 25, 64]. Our research used accessible data for climate factors and dengue cases.

Table 6 shows the accuracy of the BN classifier with TRF compared with the other models that used climate factors. All the models compared in this study used binary classification in dengue outbreak prediction. [16, 22, 48, 63] including the proposed model in this study classified dengue outbreak to “outbreak” and “no outbreak”, [21] classified it as “less” and “no case”. The proposed model with TRF achieved the highest accuracy of 92.35% compared with the other models. Besides that, this research used higher number of data compared to [21, 22] which yield an accuracy of more than 90%.

**Table 6:** Benchmarking and comparing accuracy of the proposed model with previous studies on dengue outbreak prediction model that uses accessible data

References	Year	Model	Accuracy (%)
[63]	2018	Correlation and autoregressive distributed lag model	84.90
[21]	2016	C-SVC kernel and RBF	90.50
[22]	2013	Poisson multivariate regression models	90.00
[16]	2010	Artificial neural networks	82.39
[48]	2008	Automatic prediction system by using entropy and artificial neural network	85.92
Our proposed model		Bayes network model using TRF	Accuracy = 92.35

## Discussions

In this study, the use of TRF in BN classifiers managed to outperform the accuracies obtained by other studies compared in this research. It managed to reduce the error of prediction models as well. We believed TRF is the contributing factor that enhances the accuracy. This factor is believed to retain the accuracy of the outbreak prediction model in other countries with similar geographical settings. This will definitely impact many countries such as Philippines, Indonesia, Thailand, Vietnam and Singapore that has similar geographical settings. The risk factors used by all of these countries are portrayed in Table 1. However, countries with different geographical settings, may differs in terms of lagged temperature and rainfall values. This study also supports previous studies that shows temperature and rainfall are most important risk factor that contributes to dengue outbreak.

This outbreak prediction model is expected to particularly help authorized organizations or decision makers in health organizations, governments and other concerned groups to become aware and develop improved prevention programs in the near future. An early warning system based on this model may help in surveillance and controlling the outbreak. This will ensure good reactive management intervention to be in placed effectively and efficiently to curb the epidemics. Thus, this helps communities to be prepared to face the outbreak.

Future work should explore other Malaysian dataset as well as dataset from other countries especially in using TRF by using different machine learning models. Besides that, future research should emphasize the exploration of other risk factors for predicting dengue outbreaks.

## Conclusion

We identified a new significant risk factor, called TRF, which combined the lagged average minimum temperature of 5 weeks together with the current week and lagged cumulative rainfall for 2 weeks. TRF has contributed to dengue outbreak prediction and these lagged weather variables can be useful in determining the dengue outbreak more accurately. The research managed to reveal that the use of accurate and appropriate input factors for outbreak prediction provides enhanced and precise results.

The integration of TRF into the BN model resulted in a significant accuracy of 92.35%. The results showed that using TRF in the BN model outperformed all other outbreak prediction models considered in this study. We do acknowledge although the results showed only almost 1% increase compared to without TRF, this improvement is important as it managed to predict 1 more extra outbreak in every 100 predicted outbreaks. Predicting an increase of 1% outbreak will definitely give significant impact especially for public health surveillance in dealing with infectious diseases like dengue.

Although many risk factors for dengue outbreak are available, we only focused on the detailed analysis of temperature and rain risk factors for dengue outbreaks, which have been emphasised as the most important factors due to the analysis of importance and access limitation. Future researchers should also test and explore the TRF factors in other datasets from different countries, region or different time period. This research is believed to be an eye opener for future researchers in exploring lagged variables in their outbreak prediction, which include but not limited to dengue. Besides that, the use of deep learning in dengue outbreak prediction should also be ventured.

## Data Availability

Raw Dengue confirmed cases available in portal of Ministry of Health (Malaysia): http://www.moh.gov.my/index.php/database_stores/store_view/1 For the meteorological data: 1. http://www.weather.com 2. Available upon reasonable request from the authors (processed data).

## Abbreviations

ANN: Artificial neural networks
BN: Bayes network
DLNM: Distributed lag non-linear model
GA: Genetic algorithm
GEE: Generalized estimating equation
GIS: Geographic information system
GLM: Generalised linear model
MCMC: Markov chain Monte Carlo
MMD: Malaysian Meteorological Department
MOH: Ministry of Health Malaysia
NBR: Negative binomial regression
PCC: Pearson correlation coefficient
PPMCC: Pearson product-moment correlation coefficient
RBF: Radial basis function
SRCC: Spearman’s rank correlation coefficient
SVM: Support vector machine
TRF: TempeRain factor
WHO: World Health Organization
